# *Emx2* as a novel tool to suppress glioblastoma

**DOI:** 10.18632/oncotarget.9322

**Published:** 2016-05-13

**Authors:** Carmen Falcone, Antonio Daga, Giampiero Leanza, Antonello Mallamaci

**Affiliations:** ^1^ Department of Neuroscience, SISSA, 34136 Trieste, Italy; ^2^ DIPOE, IRCCS AOU San Martino IST, 16132 Genoa, Italy; ^3^ Department of Life Sciences, University of Trieste, 34127 Trieste, Italy

**Keywords:** Emx2, glioblastoma, gene therapy, EGFR, SOX2

## Abstract

Glioblastoma is a devastating CNS tumour for which no cure is presently available. We wondered if manipulation of *Emx2*, which normally antagonizes cortico-cerebral astrogenesis by inhibiting proliferation of astrocyte progenitors, may be employed to counteract it. We found that *Emx2* overexpression induced the collapse of seven out of seven *in vitro* tested glioblastoma cell lines. Moreover, it suppressed four out of four of these lines *in vivo*. As proven by dedicated rescue assays, the antioncogenic activity of *Emx2* originated from its impact on at least six metabolic nodes, which accounts for the robustness of its effect. Finally, in two out of two tested lines, the tumor culture collapse was also achieved when *Emx2* was driven by a neural stem cell-specific promoter, likely active within tumor-initiating cells. All that points to *Emx2* as a novel, promising tool for therapy of glioblastoma and prevention of its recurrencies.

## INTRODUCTION

*Emx2* is a transcription factor gene controlling a variety of brain developmental subroutines [1]. It cooperates with *Otx2* and *Pax6* in promoting formation and proper rostro-caudal specification of prosomeres 1-4, respectively [2]. Together with *Pax6*, it specifies dorsal telencephalon as pallium [3]. Within the early developing pallium, it stimulates the expansion of the neural precursor pool by delaying neuronogenesis progression [4] and the proper generation/survival of Cajal-Retzius pioneer neurons [5]. Moreover, it promotes the development of hippocampal and visual territories [6, 7].

Recently, we found that *Emx2* also inhibits astrogliogenesis, by decreasing proliferation of astrocyte-committed progenitors via *EgfR* and *Fgf9* downregulation [8, 9]. Furthermore, several groups reported an inverse correlation between *EMX2* expression levels and malignancy of a number of non neural tumors [10–14]. These findings led us to hypothesize employing *Emx2* as a tool to suppress highly proliferating glioblastoma multiforme tumors (GBM).

GBM, also classified as WHO grade IV glioma, is the most aggressive malignant primary brain tumor in humans. It can affect cerebral cortex, cerebellum, brainstem and spinal cord. It mainly appears around 65-75 years, as a primary tumor or a recurrency of a previous, lower-grade glioma. Neurological symptoms are highly heterogeneous. Final prognosis is very poor. State of the art treatment combines surgery, temozolomide (TMZ) chemotherapy and radiation. Median survival upon this treatment is 14 months (only 4 in the absence of treatment) [15, 16].

GBMs are characterized by high mitotic rates, diminished apoptosis, poorly differentiated astrocytes and rich neoangiogenesis. Despite these commonalities, their origin and genetic features are highly heterogeneous. Nevertheless, GBMs (in particular, advanced/recurrent ones) share specific structural mutations and copy number variations, among which *EGFR* and *PDGFRA* amplification, as well as *NF1*, *PTEN* and *CDKN2A/B* loss [17].

Here we show that, in all GBM lines tested, *Emx2* overexpression suppresses glioblastoma growth, both *in vitro* and *in vivo*. Interestingly, *Emx2* activity relies on modulation of a number of malignancy-related genes, including a subset of those affected in GBM by late, oncogenic copy number variations. This may result into an appreciable therapeutic effect on a large variety of GBMs and prevent selection of drug-resistant clones as well as recurrencies. Finally, *Emx2* overexpression driven by the stem-cell-specific “*neuro-Nestin promoter*” [8] is still able to fully suppress tumor cultures. Therefore, this approach might be appropriate to target GBM cells while not damaging differentiated not-cancerous cells, and adequate to eradicate tumor-initiating-cells, so preventing GBM recurrencies.

## RESULTS

### *Emx2* overexpression kills glioblastoma cells *in vitro*

**Figure 1:**
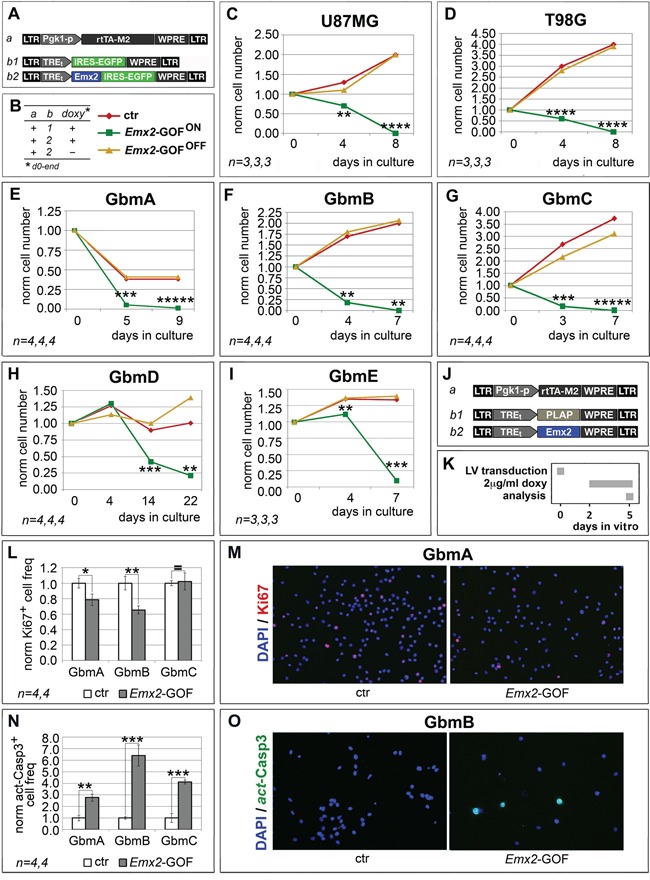
Population dynamics of *Emx2* gain-of-function GBM cultures *In vitro* kinetic progression of U87MG, T98G, GbmA, GbmB, GbmC, GbmD and GbmE GBM lines **C-I**., engineered by lentiviral vectors and TetON technology as in **A, B.**, and kept as adherent **C, D.** or floating cultures **E-I.**, under Fgf2 and Egf. Ki67^+^ proliferating **L, M.** and activated-Casp3^+^ apoptotic **N, O.** fractions of GbmA, GbmB and GbmC glioblastoma cells, engineered by control (**J.**, a-b1) and *Emx2*-GOF (J, a-b2) lentiviral sets, and kept as floating cultures according to the timetable in **K.** Cell numbers were normalized against *t*=0 values (C-I), or control values **L, N.** [As for (L, N), absolute average control cell frequencies were: 0.207, 0.155 and 0.131 (Ki67^+^, in GbmA, GbmB and GbmC cultures, respectively); 0.001, 0.012 and 0.012 (actCasp3^+^, in GbmA, GbmB and GbmC cultures, respectively)]. *n* is the number of biological replicates. *p*-value was calculated by t-test (one-tail, unpaired): *p<0.05, **p<0.01, ***p<0.001, ****p<0.0001, *****p<0.00001.

To assess if *Emx2* can antagonize glioblastoma multiforme, we overexpressed its coding sequence in 2 GBM lines (U87MG and T98G) as well as in GBM cell cultures originating from 5 different patients (GbmA, GbmB, GbmC, GbmD and GbmE), via lentiviral vectors and TetON technology. As controls, we employed the corresponding GBM cultures, infected by *Egfp*- or *Emx2*-encoding lentiviruses and kept in the presence or absence of doxycycline, respectively (Figure 1A, 1B and Supplementary Figure S1). In all cases, the activation of the *Emx2* transgene arrested the expansion of the culture and led to its collapse, usually within 7-8 days, never beyond the 22nd day (Figure 1C-1I). As we detected in *Emx2*-gain of function (-GOF) GbmA, GbmB and GbmC samples (Figure 1L-1O), this possibly reflected a decrease of the proliferating fraction (up to −34.5±5.3%, *p*<0.01, in GbmB) (Figure 1L, 1M) and an increase of the apoptotic fraction (up to 539.7±90.5%, *p*<0.001, in GbmB) (Figure 1N-1O). Intriguingly, G1-to-S phase progression was not affected (Supplementary Figure S2).

### *Emx2* antagonizes glioblastoma by a pleiotropic impact on malignancy-related processes

To cast light on molecular mechanisms underlying *Emx2* impact on GBM kinetics, we overexpressed its coding sequence in 5 GBM samples and scored mRNA levels of selected genes involved in their malignancy. These genes include: (a) a group implicated in relaying mitogenic signals along RTK cascades (*EGFR, PDGF, PDGFRA, PTEN, NF1*), (b) a group involved in the control of early G1/late G1 checkpoint (*MYC, MYCN, RB1, CDKN2A, CDKN2B, CDK4, CDK6, CCND2*), and (c) a more heterogeneous group dealing with a variety of malignancy-related processes, such as stemness, apoptosis, neovasculogenesis (*SOX2, HES1, GLI1, TRP53, MDM2, VEGF*). In all samples analyzed, *Emx2* significantly altered the expression of group (a) genes, consistently with its antioncogenic activity. It downregulated *EGFR* in all cases. In addition, it decreased *PDGF* and *PDGFRA* in 1 and 4 cases, respectively, and increased *PTEN*, in 1 case (Table 1). In a large subset of samples, *Emx2* also modulated mRNA levels of group (b) genes, again in agreement with its antioncogenic activity (Table 1). These genes include - in particular - *CDK4* and *CDK6*, mastering the early G1 checkpoint (decreased in 3 and 2 cases, respectively). Finally, *Emx2* downregulated *SOX2* in 4 samples and increased *TRP53* and *HES1* expression, in 2 and 5 samples, respectively (Table 1).

**Table 1:** Biased mRNA profiling of *Emx2* gain-of-function GBM cultures

*t*	Gbm A	Gbm B	Gbm C	Gbm D	U87-MG	gene function
	3d	3d	3d	3d	4d	
***EGFR***	0.25 ±0.06 *n=4,4; p<0.03*	0.78 ±0.03 *n=4,4; p<0.05*	0.61 ±0.04 *n=4,4; p<0.001*	0.37 ±0.13 *n=4,4; p<0.02*	0.48 ±0.02 *n=4,4; p<0.0005*	**RTK signaling**
***PDGF***	0.40 ±0.004 *n=4,4; p<0.03*	ns	ns	**1.74 ±0.12** *n=4,4; p<0.002*	ns	
***PDGFRA***	ns	0.75 ±0.11 *n=4,4; p<0.05*	0.80 ±0.07 *n=4,4; p<0.04*	0.55 ±0.01 *n=4,4; p<0.0001*	0.16 ±0.10 *n=4,4; p<0.0001*	
***PTEN***	ns	1.54 ±0.23 *n=4,4; p<0.05*	ns	>**0.30 ±0.03** *n=4,4; p<0.01*	ns	
***NF1***	ns	ns	ns	**0.26 ±0.01** *n=3,3; p<0.009*	ns	
***MYC***	ns	ns	ns	0.83 ±0.04 *n=4,4; p<0.04*	ns	**cell cycle control**
***MYCN***	0.55 ±0.03 *n=3,3; p<0.02*	ns	ns	0.40 ±0.06 *n=4,4; p<0.01*	ns	
***RB1***	ns	**0.45 ±0.16** *n=4,4; p<0.02*	ns	**0.46 ±0.04** *n=4,4; p<0.004*	ns	
***CDKN2A***	ns	2.68 ±0.37 *n=4,4; p<0.01*	ns	**0.22 ±0.01** *n=4,4; p<0.02*	ns	
***CDKN2B***	ns	2.68 ±0.37 *n=4,4; p<0.01*	ns	0.22 ±0.01 *n=4,4; p<0.02*	ns	
***CDK4***	0.48 ±0.07 *n=3,3; p<0.005*	ns	ns	0.81 ±0.06 *n=4,3; p<0.02*	0.72 ±0.08 *n=4,4; p<0.02*	
***CDK6***	0.30 ±0.02 *n=3,3; p<0.01*	ns	ns	0.36 ±0.004 *n=4,3; p<0.002*	ns	
***CCND2***	0.16 ±0.06 *n=3,3; p<0.04*	ns	ns	**1.17 ±0.03** *n=4,4; p<0.005*	ns	
***SOX2***	0.30 ±0.04 *n=4,4; p<0.02*	0.60 ±0.06 *n=4,4; p<0.03*	0.70 ±0.11 *n=4,4; p<0.04*	0.19 ±0.01 *n=4,4; p<0.00001*	ns	**other malignancy-related**
***HES1***	2.56 ±0.37 *n=3,3; p<0.008*	8.02 ±0.27 *n=4,4; p<0.0001*	1.38 ±0.05 *n=4,4; p<0.001*	1.28 ±0.02 *n=4,4; p<0.02*	4.34 ±0.80 *n=4,4; p<0.004*	
***GLI1***	ns	**2.60 ±0.65** *n=4,4; p<0.05*	ns	**2.90 ±0.25** *n=4,4; p<0.001*	ns	
***TRP53***	ns	2.87 ±0.57 *n=4,4; p<0.008*	4.58 ±0.87 *n=3,3; p<0.01*	**0.67 ±0.05** *n=4,4; p<0.001*	**0.66 ±0.08** *n=4,4; p<0.04*	
***MDM2***	ns	ns	ns	0.46 ±0.06 *n=4,3; p<0.03*	ns	
***VEGF***	ns	ns	ns	ns	ns	

mRNA levels of presumptive mediators of *Emx2* anti-oncogenic activity in GBM cells engineered as in Figure 1. Seven days after lentiviral transduction, doxycyclin was added at 2 μg/ml. RNA samples were collected at time “*t*” after doxycyclin addition. qRT-PCR results, normalized against *GAPDH* and further normalized against their own negative controls, are shown as average ± s.e.m. Values possibly accounting for *Emx2* anti-oncogenic activity are highlighted in blue. ns, not significant. *n* is the number of biological replicates. *p*-value was calculated by t-test (one-tail, unpaired).

**Figure 2:**
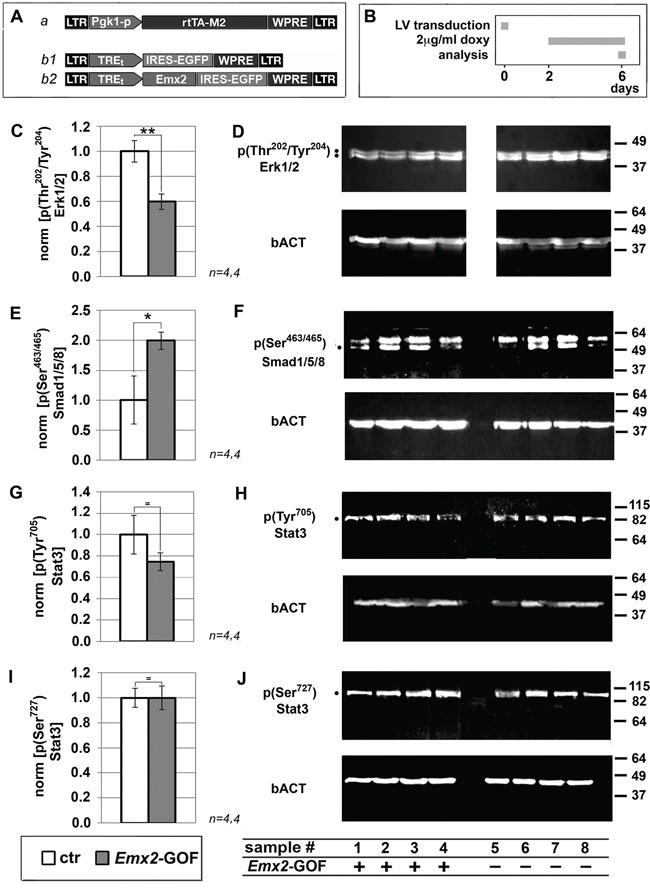
Immunoprofiling of *Emx2* gain-of-function GBM cultures for key intracellular signalling transducers Evaluation of p(Thr^202^/Tyr^204^)Erk1/2 **C, D.**, p(Ser^463^/Ser^465^)Smad1/5/8 **E, F.** p(Tyr^705^)Stat3 **G, H.** and p(Ser727)Stat3 **I,J.** levels in U87 cell samples, engineered as in **A, B.** Values were normalied against controls. *n* is the number of biological replicates. *p*-value was calculated by t-test (one-tail, unpaired): *p<0.05, **p<0.01.

To complement mRNA profiling, we also monitored *Emx2* overexpressing GBM cells for key phospho-proteins involved in malignancy-related, intracellular signal transduction (Figure 2 and Supplementary Figure S3). We found a significant decrease of p(Thr^202^/Tyr^204^)Erk1/2 (−40.3±6.3%, p<0.005, see Figure 2C, 2D). This may stem from depressed EGF and PDGF signalling. It may be a key determinant of the kinetic behaviour of *Emx2*-GOF GBM cells [16]. Furthermore, we detected a robust increase of p(Ser^463/465^)Smad1,5,8 (+100%, p<0.003, see Figure 2E, 2F). This is an index of enhanced Bmp signalling, which was shown to be instrumental to *Emx2*-dependent inhibition of astroblast proliferation [9]. Finally, we found that Stat3 phosphorylation levels in Tyr^705^ and Ser^727^, crucial to self-renewing abilities of GBM cells [18], were unchanged (Figure 2G-2J).

**Figure 3:**
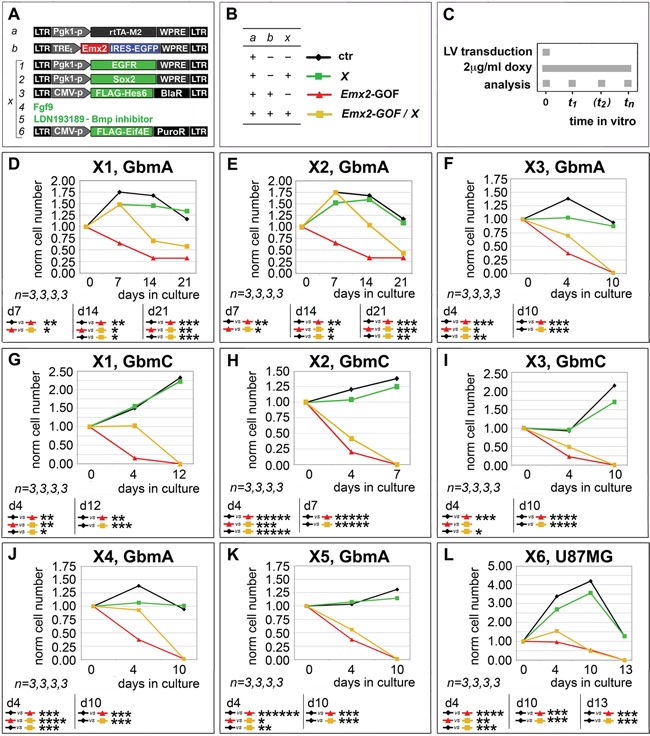
Rescue of *Emx2* antioncogenic activity via modulation of its presumptive mediators GBM cells were engineered and cultured as in **A-C.** In particular, lentiviruses harboring an *IRES-EGFP* or a *PLAP* module under the control of a *TREt* promoter were used as controls for “b” and “X1, X2, X3, X6”, respectively. Cells were scored for the capability of selected “X” agents (restoring presumptive mediators of *Emx2* anti-oncogenic activity) to rescue their control kinetic profiles **D-L.** *n* is the number of biological replicates. *p*-value was calculated by t-test (one-tail, unpaired).

Next, we tested the functional relevance of selected mRNA/protein changes described above to the *Emx2* antioncogenic activity. For this purpose, we chose a few “X” agents neutralizing such changes and evaluated their capability to rescue the original GBM kinetic profiles (Figure 3A-3C).

First, we tried to restore the basic expansion rate of GbmA and GbmC cultures, previously made gain-of-function for *Emx2*, by transducing them with an *EGFR*-expressing transgene (Figure 3A, rescue X1). This manipulation slowed down the decline of these cultures, however only in a partial and temporary fashion (Figure 3D, 3G). A similar effect was elicited by overexpression of the stemness-factor *SOX2* (Figure 3A, rescue X2) in *Emx2*-GOF GbmA and GbmC (Figure 3E, 3H). Noticeably, neither *EGFR* nor *SOX2* overexpression perturbed GBM kinetics in control conditions (Figure 3D, 3E, 3G, 3H).

Moreover, we tried to counteract *HES1*, one of the main *Emx2*-responders, whose overexpression was previously linked to proliferation arrest in a variety of contexts [19–21]. To this aim, we delivered its established functional antagonist *HES6* [22] to GBM cells (Figure 3A, rescue X3). This manipulation slowed down the collapse of GbmA and GbmC cultures overexpressing *Emx2*, while not fully preventing it (Figure 3F, 3I). Conversely, *HES6* overexpression did not promote GBM expansion in control conditions (Figure 3F, 3I).

Next, we tried to restore RTK signalling defects evoked by *Emx2* (Figure 2C, 2D), providing GBM cultures with an excess of Fgf9 (Figure 3A, rescue X4). This is a key ligand down-regulated by *Emx2* [9] and proven to promote proliferation within the astrocytic lineage [23]. Also, we silenced BMP signalling (Figure 3A, rescue X5), already proposed as a key therapeutic tool against GBM [24, 25]. Both manipulations slowed down the decline of *Emx2*-GOF GbmA cultures, however only to a partial extent (Figure 3J, 3K). Neither Fgf9 nor BMP-inhibitor delivery promoted an expansion of control GbmA cells (Figure 3J, 3K).

Finally, in addition to *Emx2* impact on transcription, we considered the possibility that the anti-oncogenic activity of this protein could be strenghtened by its capability to chelate the translational factor Eif4e [26]. In agreement with this prediction, *Eif4e* overexpression in *Emx2*-GOF U87MG cultures (Figure 3A, rescue X6) delayed their decline, while not affecting U87MG controls (Figure 3L).

All that suggests that *Emx2* may act by perturbing a number of genes and metabolic nodes crucial to GBM aggressiveness. It points at *Emx2* as a promising therapeutic tool to simultaneously attack a variety of key effectors of GBM malignancy.

### *Emx2* overexpression elicits GBM collapse *in vivo*

**Figure 4:**
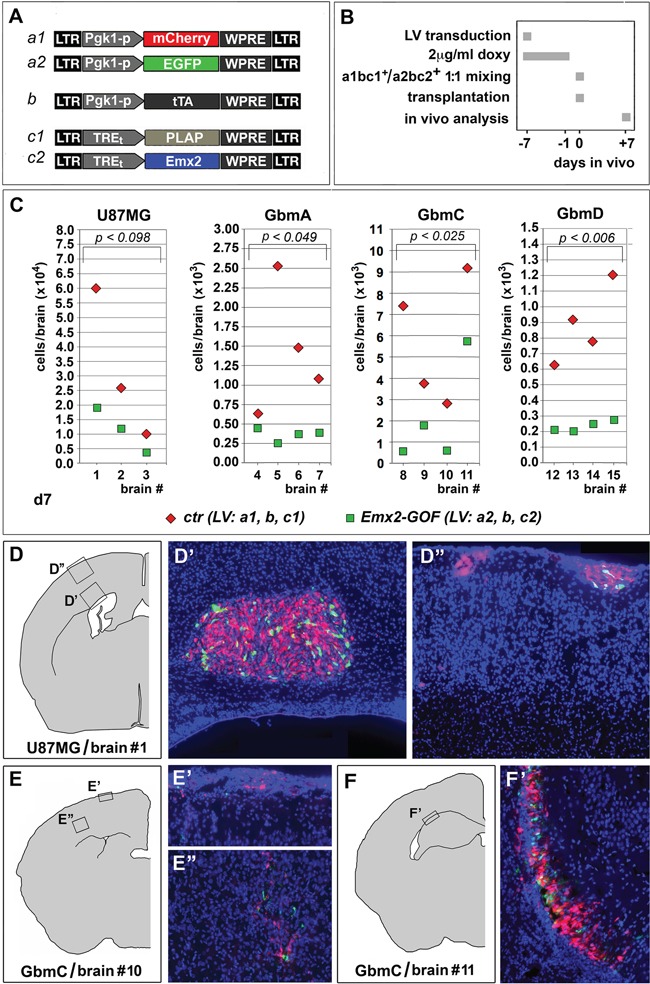
*Emx2* antioncogenic activity *in vivo* Experimental strategy and employed lentiviral vectors are shown in **A, B.** A 1:1 mix of differently fluoro-labelled, *Emx2*-GOF and control engineered GBM cells was transplanted into the cortical parenchyma of P4 wild type mouse pups. One week later, engrafted cells of different genotypes were scored in every single brain **C-F'.** 3-4 different brains were analyzed for every GBM line tested. *p*-value was calculated by t-test (one-tail, paired).

To assess the portability of *Emx2* antioncogenic activity *in vivo*, we transplanted engineered GBM cells (U87MG, GbmA, GbmC and GbmD) into the neocortical parenchyma of P4 wild-type mouse pups. Specifically, we injected a 1:1 mix of cells, made alternatively gain-of-function for *Emx2* or a control, and labelled with Egfp and mCherry, respectively. One week later, we sacrificed the animals and scored each brain for the ratio between the number of *Emx2*-GOF cells (Egfp^+^) and the number of control cells (mCherry^+^). This ratio was equal to 0.40±0.05 (*p*<0.099, *n*=3), 0.44±0.13 (*p*<0.049, *n*=4), 0.34±0.12 (*p*<0.025, *n*=4) and 0.29±0.04 (*p*<0.006, *n*=4), for U87MG, GbmA, GbmC and GbmD, respectively. This indicates that *Emx2* exerts a robust antioncogenic activity even *in vivo* (Figure 4). (In a previous pilot test run with Egfp^+^ and mCherry^+^ GBM cells not harboring additional transgenes, this ratio was close to 1) (Supplementary Figure S4).

**Figure 5:**
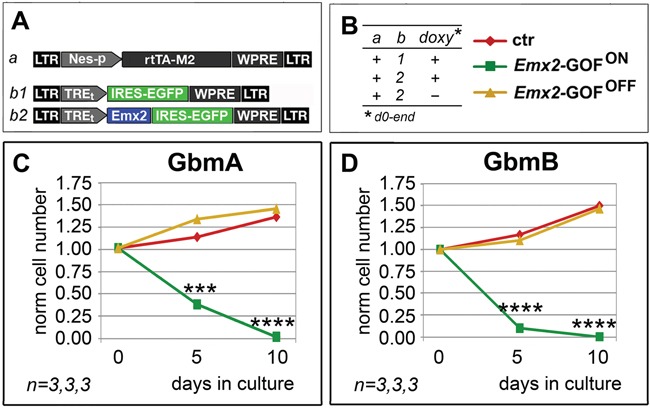
Persisting antioncogenic efficacy of *Emx2* upon neural nestin enhancer-restricted overexpression *In vitro* kinetic progression of GbmA and GbmB lines **C, D.**, engineered by lentiviral vectors and TetON technology as in **A, B.**, and kept as floating cultures, under Fgf2 and Egf. Cell numbers were normalized against *t*=0 values. *n* is the number of biological replicates. *p*-value was calculated by t-test (one-tail, unpaired): ***p<0.001, ****p<0.0001.

Disappontingly, *Emx2* overexpression in pyramidal neurons is highly toxic (our unpublished data). Therefore, generalized *Emx2* delivery to the diseased brain of GBM patients would not be a suitable approach. To circumvent this issue, we thought to restrict therapeutic *Emx2* overexpression to tumor precursor cells, by putting it under the control of a cis-active element selectively firing in neural stem cells (Figure 5A, “Nes-p”; Supplementary Figure S5). Remarkably, this design turned out to be feasible, as it successfully replicated the kinetic outcome elicited upon generalized *Emx2* overexpression (compare Figure 5C, 5D and Figure 1E, 1F).

## DISCUSSION

Here we showed that *Emx2* overexpression in a number of GBM cultures forced them to collapse, by promoting cell death and inhibiting cell proliferation. *Emx2* impact on GBM metabolism was complex and a number of genes and pathways sensitive to its overexpression were co-involved in its antitumoral activity. Remarkably, such activity was confirmed *in vivo*, upon transplantation of conditionally engineered tumor cells into the neocortical parenchyma of mouse neonates. Last but not least, restricting *Emx2* overexpression to presumptive tumor stem cells replicated the outcome of generalized gene overexpression.

Multiple bodies of correlative data suggest that *EMX2* downregulation could contribute to the genesis of GBMs. The COSMIC database (http://cancer.sanger.ac.uk/cosmic) reports two distinct homozygous *EMX2* deletions occurring in 2 out of 801 gliomas (data not shown). Analysis of Allen Brain - Ivy Glioblastoma Atlas data showed us that *EMX2*-mRNA levels are specifically reduced by ≥2-folds in GBM tumors with respect to surrounding tissue (Supplementary Figure S6). Next, in the majority of glioblastoma cultures analyzed in the present study, endogenous *EMX2*-mRNA was undetectable. When it was present, its level was lower than in astrogenic, fetal cortico-cerebral NSCs (Supplementary Figure S7). Finally, a consistent scenario was found in acutely immunopanned GBM astrocytes, compared to astrocytes purified from the surrounding healthy tissue [27]. Albeit intriguing, all these correlative data are obviously not sufficient to draw firm conclusions about *EMX2* role in GBM etiopathogenesis, which was out of the aims of the present study. Nevertheless, our data suggest that, regardless of its role in the oncogenic process, *Emx2* may be a powerful tool for counteracting GBM tumors.

Subject of this study were two established GBM cell lines (U87MG and T98G) and five tumor cultures derived from operated GBM patients. Promisingly, all of them robustly responded to *Emx2* and rapidly collapsed, because of defective proliferation and exaggerated cell death (Figure 1 and Supplementary Figure S2).

Noticeably, their molecular responses were complex and not stereotyped (Table 1), possibly reflecting GBM etiopathogenetic heterogeneity [28, 17, 29]. The vast majority of molecular changes evoked by *Emx2* were consistent with its antitumor activity (Table 1 and Figure 2), a selection of them was proven to be instrumental to it (Figure 3). However, in no case, counteracting each of these changes could fully restore original kinetic properties of the cultures (Figure 3). All this means that *Emx2* antioncogenic efficacy may emerge as a consequence of its ability to attack a *variety* of metabolic nodes crucial to malignancy. In addition to a robust inhibition of GBM expansion, this ability might help preventing selection of drug-resistant clones and recurrencies. Intriguingly, a number of *Emx2*-repressed and -stimulated genes reported above are the very same affected by duplications and deletions in late stage glioma cancers, respectively [17]. This further suggests that our manipulation could be therapeutically effective on a variety of high grade gliomas, regardless of their primary molecular origin.

Remarkably, *Emx2* overexpression elicited a pronounced anti-GBM activity even *in vivo* (Figure 4). This was found by cotransplanting conditionally engineered tumor cells, alternatively expressing *Emx2* or a control transgene, into the cortical parenchyma of wild type mouse pups and scoring the outcome one week later. This is a novel experimental setup, allowing to preliminarly assess antioncogenic power *in vivo*, quickly, in an immunocompetent environment, and in the presence of strong progliogenic cues. Finally, of particular therapeutic interest is the fact that a collapse of engineered GBM cultures also occurred when *Emx2* overexpression was restricted to Nes-p^+^ precursor cells (Figure 5). These cells, in fact, are likely to include tumor initiating cells (TICs), from which tumor recurrencies are supposed to originate [38]. Such cells may escape even the attack by the most advanced oncolytic vectors developed against GBM [39].

All these results point to *Emx2* as a novel, promising tool for GBM therapy. However, for this purpose long-term survival tests as well as the selection of a more appropriate, not-genotoxic vectors for gene delivery, are mandatory. Moreover, an in depth exploration of mechanisms mediating *Emx2* activity, by unbiased GBM transcriptome profiling, is also due. These issues will be hopefully subject of a dedicated follow-up study.

## MATERIALS AND METHODS

### Lentiviral vectors packaging and titration

All lentiviruses were generated and titrated as previously described [40]. The full list of genomic plasmids used for lentiviruses packging is reported in Supplementary Materials.

### GBM cell culture

Human U87MG GBM cell line (derived from a malignant glioma from a female patient by explant technique; PCR-validated as follows: Amelogenin: X; CSF1PO: 10,11; D13S317: 8,11; D16S539: 12; D5S818: 11,12; D7S820: 8,9; THO1: 9.3; TPOX: 8; vWA: 15,17) and T98G GBM cell line (derived from glioblastoma multiform tumour from a 61-year-old Caucasian male; PCR-validated as follows: Amelogenin: X,Y; CSF1PO: 10,12; D13S317: 13; D16S539: 13; D5S818: 10,12; D7S820: 9,10; THO1: 7,9.3; TPOX: 8; vWA: 17,20) were purchased from SIGMA (#89081402 and #92090213, respectively). Low passage criopreserved samples of them were employed to run this analysis. They were kept as adherent cultures in DMEM/Glutamax medium (ThermoFisher, Waltham - MA, #31966), supplemented with 10% FBS and 1X Pencillin/Streptomycin. Cells were cultured at 500 cells/μl and passaged by trypsin on days of counting or, at most, every 4 days.

GbmA-E cells originate from GBM surgical samples collected at IRCCS A.O.U. San Martino-IST, Dept of Neuroscience and Sense organs, Unit of Neurosurgery and Neurotraumatology, with patients' informed consent and in compliance with pertinent Italian law. They were derived in AD laboratory. Low passage criopreserved samples of them were employed in AM laboratory to run this analysis. GbmA cells were cultured in “DMEM / F12 / glutamax / NeurobasalA” (ThermoFisher #10888-022). GbmB-E were cultured as spheres in NeuroCult™ NS-A Proliferation Kit (Human) (StemCell Technologies, Vancouver - Canada, #05751). In both cases, mediums were supplemented with 1X Penicillin/Streptomycin, 2 μg/ml human heparin (StemCell Technologies #07980), 20 ng/ml recombinant human EGF (ThermoFisher #PHG0311), 20 ng/ml recombinant human FGF2 (ThermoFisher #PHG0261). All the cells were cultured under normoxic conditions (5% CO2, 21% O2, 74% N2). Cells were cultured at 500 cells/μl and passaged by Accutase (Sigma, Milan - Italy, #A6964) on days of counting or, at most, every 4 days.

When required, cells were acutely infected, at a concentration of 500 cells/μl, by a mix containing lentiviral vectors, each one at a multiplicity of infection (m.o.i) = 6. This moi is sufficient to infect the almost totality of GBM cells in such conditions. Where required, they were subsequently transferred to polylysinated coverslips. In specific cases, medium was supplemented by 0.7 μM LDN193189 (Stemgent, Lexington - MA, #130-096-226), 20 ng/ml Fgf9 (Sigma #SRP4057-10UG), or 10μg/ml BrdU. TetON-controlled transgenes were switched on by 2 μg/ml doxycyclin (Sigma #D9891-10G). *n* is the number of biological replicates. *p*-value was calculated by t-test (one-tail, unpaired).

### Cell growth curves

After sphere dissociation, 2×10^5^ GBM cells/well were seeded in a 24-well plate and infected with LV_Pgk1p-rtTA-M2-WPRE and LV_TREt-IRES-EGFP-WPRE or LV_TREt-Emx2-IRES-EGFP-WPRE, each at m.o.i. 6. Viable cells (trypan blue-excluding) were counted at fixed days on a hemocytometer. After every cell count, differently engineered cells were plated at the same concentration (2 × 10^5^ GBM cells/well). Cell counting was performed on 1/200 of each biological sample (in case of Figure 1, 3 and 5 data, at *t*=0, each sample included 200,000 cells). Growth curves were interrupted when *Emx2*-GOF cell cultures had collapsed.

### mRNA profiling

Aliquots of 2*10^5^ GBM cells were infected with a lentivector mix containing LV_Pgk1p-rtTA-M2-WPRE and LV_TREt-IRES-EGFP-WPRE or LV_TREt-Emx2-IRES-EGFP-WPRE, each at m.o.i. 6. Seven days after infection, 2 μg/ml doxycycline was added. Three or four days later, cell pellets were processed for RNA extraction by Trizol™ (ThermoFisher #15596-026). Agarose gel electrophoresis and spectrophotometric measurements (NanoDrop ND-1000) were employed to estimate quantity, quality and purity of the resulting RNA. Prior to analysis, samples were processed by the TurboDNAfree kit (ThermoFisher #AM1907), according to manufacturer's instructions.

At least 1.0 μg RNA from each sample was retrotranscribed by SuperScriptIII™ (ThermoFisher #18080044) in the presence of random hexamers, according to manufacturer's instructions. RT-minus samples were scored as controls, in the case of intronless transcripts.

1/50 of the resulting cDNA was used as substrate of any subsequent qPCR reaction. PCR reactions were performed by the SybrGreen™ platform (Biorad, Milan - Italy, #1725270), according to manufacturer's instructions.

The full list of primers is reported in Supplementary Materials and Methods.

Each biological replicate was scored at least in technical triplicate and data were normalized against *GAPDH*. Results were averaged and further normalized on their controls. Averages ± s.e.m.'s were reported in Table 1. Statistical significance of results was evaluated by the t-test (one-tail; unpaired). “*n*” is the number of samples.

### Western blots

Western analysis was performed according to standard methods. Total cell lysates in CHAPS buffer were quantified by BCA protein assay kit (ThermoFisher #10678484) and denatured at 95°C for 5 min, prior to loading. Thirty micrograms of proteins were loaded per each lane and run on a 12% acrylamide − 0.1% SDS gel.

Full details about antibodies employed are reported in Supplementary Materials and Methods. Different antigens and bACT were sequentially revealed by an ECL kit (GE Healthcare, Milan - Italy, #GERPN2109). Images were acquired by an Alliance LD2–77.WL apparatus (Uvitec, Cambridge) and analyzed by Adobe Photoshop CS2 software™ and Microsoft Excel 11 software™.

### Animal handling

Animal handling and subsequent procedures were in accordance with European [European Communities Council Directive of November 24, 1986 (86/609/EEC)] and Italian laws (D.L. 04.03.2014, n°26) and were approved by SISSA Board for Animal Welfare. Wild type mice (strains CD1 purchased from Harlan-Italy) were maintained at the SISSA mouse facility. P0 stands for the animal birthday.

### Cell transplantation

P4 pups were anaesthetized on ice for 40-60s. 2.0 μl of a solution containing 200,000 cells (100,000 *Emx2*-GOF-Egfp^+^ and 100,000 control-mCherry^+^ cells, pre-engineered by lentiviral transduction 1 week before), mixed with 0.02% fast-green dye in NeuroCult™ NS-A Proliferation (Human) medium (StemCell Technologies #05751), were injected through the skull into the corticocerebral parenchyma, by free hands, using a sharp pulled micropipette (hole external diameter about 40 μm) with the help of light fibers. Animals were left to recover in a warm clean cage. Then they were transferred to their mother and were finally sacrificed 7 days later.

### Sample preparation for immunofluorescence

As for the brains, they were fixed by immersion in 4% PFA overnight at 4°C, washed by 1X PBS 3 times, equilibrated in 30%sucrose-1XPBS at 4°C, included into OCT (Killik) and frozen at −80°C. Finally they were coronally sliced at 16 μm, by a Microm cryostat.

As for dissociated cell cultures, they were acutely attached on coverslips, fixed in 4% PFA for 15 min at room temperature and washed by 1X PBS 3 times.

### Immunofluorescence

Immunocytofluorescence was performed as previously described [8, 41]. Specifically, BrdU unmasking was performed by 0.2 M HCl, for 15 min at RT. The full list of antibodies employed is reported in Supplementary Materials and Methods.

Immunos were photographed on a Nikon Eclipse *TI* microscope equipped with a 20X objective and a Hamamatsu camera. Images were imported and analyzed by Photoshop CS2 (Adobe) software. Where not otherwise stated, each experiment was performed at least in biological triplicate.

For each dissociated cell culture sample, cells were counted from at least 20 randomly assorted photographic fields, by an operator blind of sample identity. As for Ki67 and BrdU quantification, 2,000 cells/sample were scored. As for actCasp3 quantification, 10,000, 2,000 and 2,000 cells/sample were scored, in case of GbmA, -B and -C cultures, respectively. Frequencies of immunoreactive cells were averaged and s.e.m.'s were calculated. Results were normalized against controls. Their statistical significance was evaluated by the t-test (one-tail; unpaired). “*n*” is the number of samples.

Pictures of transplanted brains were electronically encrypted by random inversion of green and red signals and analyzed by an operator blind of sample identity. For each brain, tumor cells were counted from 1 every 5 sections and total, Egfp^+^ and mCherry^+^ cell numbers were plotted. Statistical significance of results was evaluated by the t-test (one-tail; paired). “*n*” is the number of samples.

## SUPPLEMENTARY MATERIALS FIGURES


