# Supplementary material for: Emx2 as a novel tool to suppress glioblastoma
Source: Oncotarget. 2016 May 13;7(27):41005–16. doi: 10.18632/oncotarget.9322 (PMC5173038; doi:10.18632/oncotarget.9322)
Supplement: Supplementary file 1 [file oncotarget-07-41005-s001.pdf]

Emx2 as a novel tool to suppress glioblastoma

SUPPLEMENTARY MATERIALS

SUPPLEMENTARY DISCUSSION

Among genes mediating the antioncogenic *Emx2* activity there is *EGFR* (Figure 3D, 3G), which is implicated with *PDGFR* in transmission of mitogenic signals along RTK cascades and acts as one of the most powerful enhancers of GBM malignancy [30]. *Emx2* activity was also mediated by modulation of two stemness-related genes, *SOX2* (Figure 3E, 3H) and *HES1* (Figure 3F, 3I). The former is a key NSC promoter [31], also involved in a reciprocal excitatory loop with *EGFR* [32]. Its downregulation was expected. Conversely, upregulation of well known NSC promoter *HES1* [33] and its relevance to *Emx2* antioncogenic activity were somehow unforeseen. They could reflect the fact that *HES1*, albeit necessary to NSC self-renewal, may block cell proliferation if expressed above a given threshold [19]. Consistently, while partially restoring expansion of *Emx2*-GOF cultures, *HES6*-mediated blockade of *HES1* activity led to a reduced expansion of control cultures (Figure 3F, 3I). Reasonably *EGFR* downregulation by *Emx2* could result in depressed RTK signalling and reduced pErk levels, leading to impaired proliferation and survival. Fgf9could just fix GBM expansion (Figure 3J), by alternatively restoring pErk levels via FGFR pathway, similar to functional replacement of EGFR signalling by PDGFR and ERBBs [30]. Intriguingly, however, *Fgf9*-mRNA is barely detectable in control tumors employed in this study (not shown), suggesting that here Fgf9 possibly vicariates some of its paralogues.

Bmp capability to partially mediate *Emx2* anti-oncogenic activity (Figure 3K) was expected. In fact, Bmp signalling is a key promoter of neural differentiation and apoptosis in normal CNS development [34, 35]. Moreover, it downregulates the antiapoptotic gene BCL2 [36] and inhibits the oncogenic power of GBM-initiating cells [24].

In a large subset of cases, the impact of Emx2 on the above mentioned genes was likely due to direct transcriptional repression, as suggested by in silico prediction of evolutionarily conserved, EMX2-binding sites in their promoters (Supplementary Figure S8). Conversely, the ability of *Eif4e* to promote to some extent expansion of *Emx2*-GOF GBM cultures (Figure 3L) supports the emerging concept that a number of “transcription factors” do also act as translational regulators [37].

SUPPLEMENTARY MATERIALS AND METHODS

Murine cell cultures

They were established form E12.5 pallial precursors as described in ref [1].

Lentiviral vectors packaging and titration

Genomic plasmids used for lentiviruses packging were:

- LV_Pgk1p-rtTA-M2-WPRE, aka LV:pPgk1-rtTA2^{S}-M2 [2];

- LV_TREt-IRES-EGFP-WPRE, aka LV:TREt-IRES2-EGFP [1];

- LV_TREt-Emx2-IRES-EGFP-WPRE, aka TREt-Emx2-IRES2-EGFP [3];

- LV_TREt-IRES-PLAP-WPRE, obtained by replacing the XhoI/SalI fragment of LV_Pgk1p-EGFP-WPRE (see below) by an XhoI-compatible/SalI-compatible element, including the XbaI-AgeI 0.35kb TREt fragment of P199 [4] and the EcoRI/SalI 2.2kb IRES-PLAP fragment from pCLE [5];

- LV_TREt-Emx2-WPRE, aka LV:TREt-Emx2 [6];

- LV_Pgk1p-EGFR-WPRE, aka *pCCLsin.PPT.hPGK.EGFR.pre* [7];

- LV_Pgk1p-Sox2-WPRE, aka TetO-FUW-sox2 (Addgene #20326) [8];

- LV_Pgk1p-FLAG-Hes6-BlaR [9];

- LV_CMVp-FLAG-Eif4E (Addgene #38239) [10];

- LV_pLenti6V5D(TOPO) [9];

- LV_Pgk1p-mCherry-WPRE, constructed by transferring the mCherry module form LV_pTa1-

mCherry [3] into LV_pPgk1-EGFP-WPRE (see below), in place of EGFP;

- LV_Pgk1p-EGFP-WPRE, aka pCCLsin.PPT.hPGK.EGFP.Wpre [11];

- LV_Pgk1p-tTA-WPRE, obtained by transferring the BamHI/XhoI-cut tTA-cds fragment from LV:pTYF-1xSYN-tTA (Addgene #19980) [12] into the BamHI/SalI-cut LV_pPgk1-EGFP-WPRE, in place of EGFP;

- LV_Nesp-rtTA-M2-WPRE, aka pNes/hsp68-rtTA2[S]-M2 [3].

mRNA profiling

Primers employed in qRTPCR analysis were as follows:

- *GAPDH,* hGAPDH/Fw, 5’ CAT CAC CAT CTT CCA GGA GCG AGA TCC 3’, and hGAPDH/Rv, 5’ CAA ATG AGC CCC AGC CTT CTC CAT GG 3’

- *EGFR,* hEGFR/Fw, 5’ GAG ACC CCC AGC GCT ACC TTG TCA TTC A 3’, and hEGFR/Rv, 5’ CCA CCA CGT CGT CCA TGT CTT CTT CAT CCA 3’

- *PDGF,* hPDGF/Fw, 5’ CCT GCC CAT TCG GAG GAA GAG AAG CA 3’, and hPDGF/Rv, 5’ GGT GCA GCG TTT CAC CTC CAC GCA CG 3’

- *PDGFR,* hPDGFR/Fw, 5’ ACA CTT GCT ATT ACA ACC ACA CTC AGA CAG AAG 3’, and hPDGFR/Rv, 5’ TCC TCC ACG ATG ACT AAA TAA TCC GTC ATT CCT A 3’

- *PTEN,* hPTEN/Fw, 5’ GTT TGT GGT CTG CCA GCT AAA GGT GAA GAT A 3’, and hPTEN/Rv, 5’ CAC AGG TAA CGG CTG AGG GAA CTC AAA G 3’

- *NF1,* hNF1/Fw, 5’ AGG ACC TGA AGG TAT TCC ACA ATG CTC TCA A 3’, and hNF1/Rv, 5’ CTG AAG TTA CTT GGA CAG CAG TAG AAC CAA C 3’

- *MYC,* hMYC/Fw, 5’ CCC TGG TGC TCC ATG AGG AGA CAC 3’, and hMYC/Rv, 5’ AGG AGC CTG CCT CTT TTC CAC AGA AAC A 3’

- *MYCN,* hMYCN/Fw, 5’ GAG AGG ACA CCC TGA GCG ATT CAG ATG 3’, and hMYCN/Rv, 5’ TGG TGA ATG TGG TGA CAG CCT TGG TGT TG 3’

- *RB1,* hRB1/Fw, 5’ GAG GGA ACA TCT ATA TTT CAC CCC TGA AGA GTC 3’, and hRB1/Rv, 5’ CAG AAG TCC CGA ATG ATT CAC CAA TTG ATA CTA AGA 3’

- *CDKN2a/b,* hCDKN2a/b/Fw, 5’ CTC CAC GGC GCG GAG CCC AAC T 3’, and hCDKN2a/b/Rv, 5’ GCA GCA CCA CCA GCG TGT CCA GGA A 3’

- *CDK4,* hCDK4/Fw, 5’ GCA TCC CAA TGT TGT CCG GCT GAT GGA 3’, and hCDK4/Rv, 5’ GGT CTA CAT GCT CAA ACA CCA GGG TTA CC 3’

- *CDK6,* hCDK6/Fw, 5’ GCA CCC CAA CGT GGT CAG GTT GTT TGA TG 3’, and hCDK6/Rv, 5’ GGT CAA GTC TTG ATC GAC ATG TTC AAA CAC TAA A 3’

- *CCND2,* hCCND2/Fw, 5’ CCT GCA GCA GTA CCG TCA GGA CCA A 3’, and hCCND2/Rv, 5’ TCA CAG GTC GAT ATC CCG CAC GTC TGT A 3’

- *SOX2,* hSOX2/Fw, 5’ CGG CAC GGC CAT TAA CGG CAC ACT G 3’, and hSOX2/Rv, 5’ GTT TTC TCC ATG CTG TTT CTT ACT CTC CTC TTT TG 3’

- *TRP53,* hTRP53/Fw, 5’ CCT CCT CAG CAT CTT ATC CGA GTG GAA G 3’, and hTRP53/Rv, 5’ CAT AGG GCAC CAC CAC ACT ATG TCG AAA AG 3’

- *MDM2,* hMDM2/Fw, 5’ GTA TAA GTG TCT TTT TGT GCA CCA ACA G 3’, and hMDM2/Rv, 5’ TGT ACC TAC TGA TGG TGC TGT AAC CAC C 3’

- *GLI1,* hGLI1/Fw, 5’ GGA GGA AAG CAG ACT GAC TGT GCC AGA 3’, and hGLI1/Rv, 5’ CAG ACC AGT GCC AGC AAT GCA AGG TCC 3’

- *HES1,* hHES1/Fw, 5’ CCA AAG ACA GCA TCT GAG CAC AGA AAG TCA TC 3’, and hHES1/Rv, 5’ GCG AGC TAT CTT TCT TCA GAG CAT CCA AAA TC 3’

- *VEGF,* hVEGF/Fw, 5’ GAA GAT GTA CTC GAT CTC ATC AGG GTA C 3’, and hVEGF/Rv, 5’ CAG AAG GAG GAG GGC AGA ATC ATC AC 3’

Immunofluorescence

The following primary antibodies were used: anti-activeCaspase3, rabbit monoclonal clone C92-605 (BD Pharmingen, Milan - Italy, #559565), at 1:300; anti-BrdU, mouse monoclonal, B44 antibody (Becton-Dickinson #347580), at 1:50; anti-Tubb3 (TUJ1) mouse monoclonal (Biolegend, San Diego - CA-USA, #MMS-435P), at 1:1,000; anti-GFAP, rabbit polyclonal (DAKO #Z0334), at 1:500; anti-GFP, chicken polyclonal (Abcam #13970), at 1:400; anti-Ki67, mouse monoclonal (BD Pharmingen #550609), at 1:50; anti-RFP, rat monoclonal 5F8 (Chromotek, Planegg - Germany, #5f8-20), at 1:500; anti-Pax6, rabbit polyclonal (Biolegend #PRB-278P), at 1:300.

The following secondary antibodies were used: Alexa408 Goat Anti-Rabbit; Alexa594 Goat Anti-Mouse, Alexa488 Goat Anti-Chicken.

Western blots

The following primary antibodies were used: anti-Emx2, mouse monoclonal, M06-4F7 antibody (Abnova #H00002018-M06), at 1:300; anti-p(Thr^{202}/Tyr^{204})Erk1/2, rabbit monoclonal (Cell Signaling Technology, Leiden - The Netherlands, #4370), at 1:2000; anti-p($Ser^{463/465}$)Smad1/5/8, rabbit polyclonal (Merck-Millipore, Vimodrone - Italy, #ab3848), at 1:500; anti-p(Tyr^{705})Stat3, rabbit monoclonal (Cell Signaling Technology #9145), at 1:1000; anti-p(Ser^{727}) Stat3, rabbit polyclonal (Santa Cruz, Dallas - TX, sc-8001-R), at 1:800.

A secondary HRP-conjugated anti-rabbit antibody (ThermoFisher #32260) was used at 1:2000.

bACT was straightly detected by a peroxydase C-conjugated mouse monoclonal antibody (Sigma #A3854), used at 1:10,000.

Primary antibodies used in western blot assays were previously validated as follows:

antibody	key reference
anti-p(Thr^{202}/Tyr^{204})Erk1/2, rabbit monoclonal (Cell Signaling Technology, Leiden - The Netherlands, #4370)	Rohani MG, DiJulio DH, An JY, Hacker BM, Dale BA, Chung WO. PAR1- and PAR2-induced innate immune markers are negatively regulated by PI3K/Akt signaling pathway in oral keratinocytes. BMC Immunol. 2010;11:53.
anti-p($Ser^{463/465}$)Smad1/5/8, rabbit polyclonal (Merck-Millipore, Vimodrone, Italy, #ab3848)	Wang Q, Zhao G, Xing S, Zhang L, Yang X. Role of bone Morphogenetic proteins in form-deprivation Myopia sclera. Molecular vision 2011;17:647-657. and http://www.merckmillipore.com/IT/it/product/Anti-phospho-Smad1Smad5Smad8-Antibody,-phospho-specific-%28Ser463465%29,MM_NF-AB3848
anti-p(Tyr^{705})Stat3, rabbit monoclonal (Cell Signaling #9145)	Anand S, Stedham F, Gudgin E, Campbell P, Beer P, Green AR, Huntly BJ. Increased basal intracellular signaling patterns do not correlate with JAK2 genotype in human myeloproliferative neoplasms. Blood 2011;118:1610-1621.
anti-p(Ser^{727})Stat3, rabbit polyclonal (Santa Cruz, Dallas - TX, sc-8001-R)	Sandur SK, Pandey MK, Sung B, Aggarwal BB.5-hydroxy-2-methyl-1,4-naphthoquinone, a vitamin K3 analogue, suppresses STAT3 activation pathway through induction of protein tyrosine phosphatase, SHP-1: potential role in chemosensitization. Mol. Cancer Res. 2010;8:107-118.
anti-Emx2 mouse monoclonal, M06-4F7 antibody (Abnova #H00002018-M06)	Falcone C, Filippis C, Granzotto M, Mallamaci A Emx2 expression levels in NSCs modulate astrogenesis rates by regulating EgfR and Fgf9. Glia. 2015; 63:412–22.

SUPPLEMENTARY REFERENCES

1. Furnari FB, Cloughesy TF, Cavenee WK, Mischel PS. Heterogeneity of epidermal growth factor receptor signalling networks in glioblastoma. Nat Rev Cancer. 2015;15:302–10.
2. Gangemi RM, Griffero F, Marubbi D, Perera M, Capra MC, Malatesta P, Ravetti GL, Zona GL, Daga A, Corte G. SOX2 silencing in glioblastoma tumor-initiating cells causes stop of proliferation and loss of tumorigenicity. Stem Cells. 2009;27:40–8.
3. Hu Q, Zhang L, Wen J, Wang S, Li M, Feng R, Yang X, Li L. The EGF receptor-sox2-EGF receptor feedback loop positively regulates the self-renewal of neural precursor cells. Stem Cells. 2010;28:279–86.
4. Ohtsuka T, Sakamoto M, Guillemot F, Kageyama R. Roles of the basic helix-loop-helix genes Hes1 and Hes5 in expansion of neural stem cells of the developing brain. J Biol Chem. 2001;276:30467–74.
5. Baek JH, Hatakeyama J, Sakamoto S, Ohtsuka T, Kageyama R. Persistent and high levels of Hes1 expression regulate boundary formation in the developing central nervous system. Development. 2006;133:2467–76.
6. Furuta Y, Piston DW, Hogan BL. Bone morphogenetic proteins (BMPs) as regulators of dorsal forebrain development. Development. 1997;124:2203–12.
7. Sun Y, Nadal-Vicens M, Misono S, Lin MZ, Zubiaga A, Hua X, Fan G, Greenberg ME. Neurogenin promotes neurogenesis and inhibits glial differentiation by independent mechanisms. Cell. 2001;104:365–76.
8. Liu B, Chen Q, Tian D, Wu L, Dong H, Wang J, Ji B, Zhu X, Cai Q, Wang L, Zhang S. BMP4 reverses multidrug resistance through modulation of BCL-2 and GDNF in glioblastoma. Brain Research. 2013;1507:115–24.
9. Piccirillo SG, Reynolds BA, Zanetti N, Lamorte G, Binda E, Broggi G, Brem H, Olivi A, Dimeco F, Vescovi AL. Bone morphogenetic proteins inhibit the tumorigenic potential of human brain tumour-initiating cells. Nature. 2006;444:761–5.
10. Rezsohazy R. Non-transcriptional interactions of Hox proteins: inventory, facts, and future directions. Dev Dyn. 2014;243:117–31.
11. Falcone C, Filippis C, Granzotto M, Mallamaci A. Emx2 expression levels in NSCs modulate astrogenesis rates by regulating EgfR and Fgf9. Glia. 2015;63:412–22.
12. Spigoni G, Gedressi C, Mallamaci A. Regulation of Emx2 expression by antisense transcripts in murine cortico-cerebral precursors. PLoS ONE. 2010;5:e8658.
13. Brancaccio M, Pivetta C, Granzotto M, Filippis C, Mallamaci A. Emx2 and Foxg1 inhibit gliogenesis and promote neuronogenesis. Stem Cells. 2010;28:1206–18.
14. Stegmeier F, Hu G, Rickles RJ, Hannon GJ, Elledge SJ. A lentiviral microRNA-based system for single-copy polymerase II-regulated RNA interference in mammalian cells. Proc Natl Acad Sci USA. 2005;102:13212–7.
15. Gaiano N, Nye JS, Fishell G. Radial glial identity is promoted by Notch1 signaling in the murine forebrain. Neuron. 2000;26:395–404.
16. Raciti M, Granzotto M, Duc MD, Fimiani C, Cellot G, Cherubini E, Mallamaci A. Reprogramming fibroblasts to neural-precursor-like cells by structured overexpression of pallial patterning genes. Mol Cell Neurosci. 2013;57:42–53.
17. Mazzoleni S, Politi LS, Pala M, Cominelli M, Franzin A, Sergi Sergi L, Falini A, De Palma M, Bulfone A, Poliani PL, Galli R. Epidermal Growth Factor Receptor Expression Identifies Functionally and Molecularly Distinct Tumor-Initiating Cells in Human Glioblastoma Multiforme and Is Required for Gliomagenesis. Cancer Research. 2010;70:7500–13.
18. Brambrink T, Foreman R, Welstead GG, Lengner CJ, Wernig M, Suh H, Jaenisch R. Sequential expression of pluripotency markers during direct reprogramming of mouse somatic cells. Cell Stem Cell. 2008;2:151–9.
19. Hartman J, Lam EW-F, Gustafsson J-Å, Ström A. Hes-6, an inhibitor of Hes-1, is regulated by 17β-estradiol and promotes breast cancer cell proliferation. Breast Cancer Research. 2009;11:R79.
20. Thoreen CC, Chantranupong L, Keys HR, Wang T, Gray NS, Sabatini DM. A unifying model for mTORC1-mediated regulation of mRNA translation. Nature. 2012;485:109–13.
21. Follenzi A, Naldini L. HIV-based vectors. Preparation and use. Methods Mol Med. 2002;69:259–74.
22. Liu B, Wang S, Brenner M, Paton JFR, Kasparov S. Enhancement of cell-specific transgene expression from a Tet-Off regulatory system using a transcriptional amplification strategy in the rat brain. J Gene Med. 2008;10:583–92.

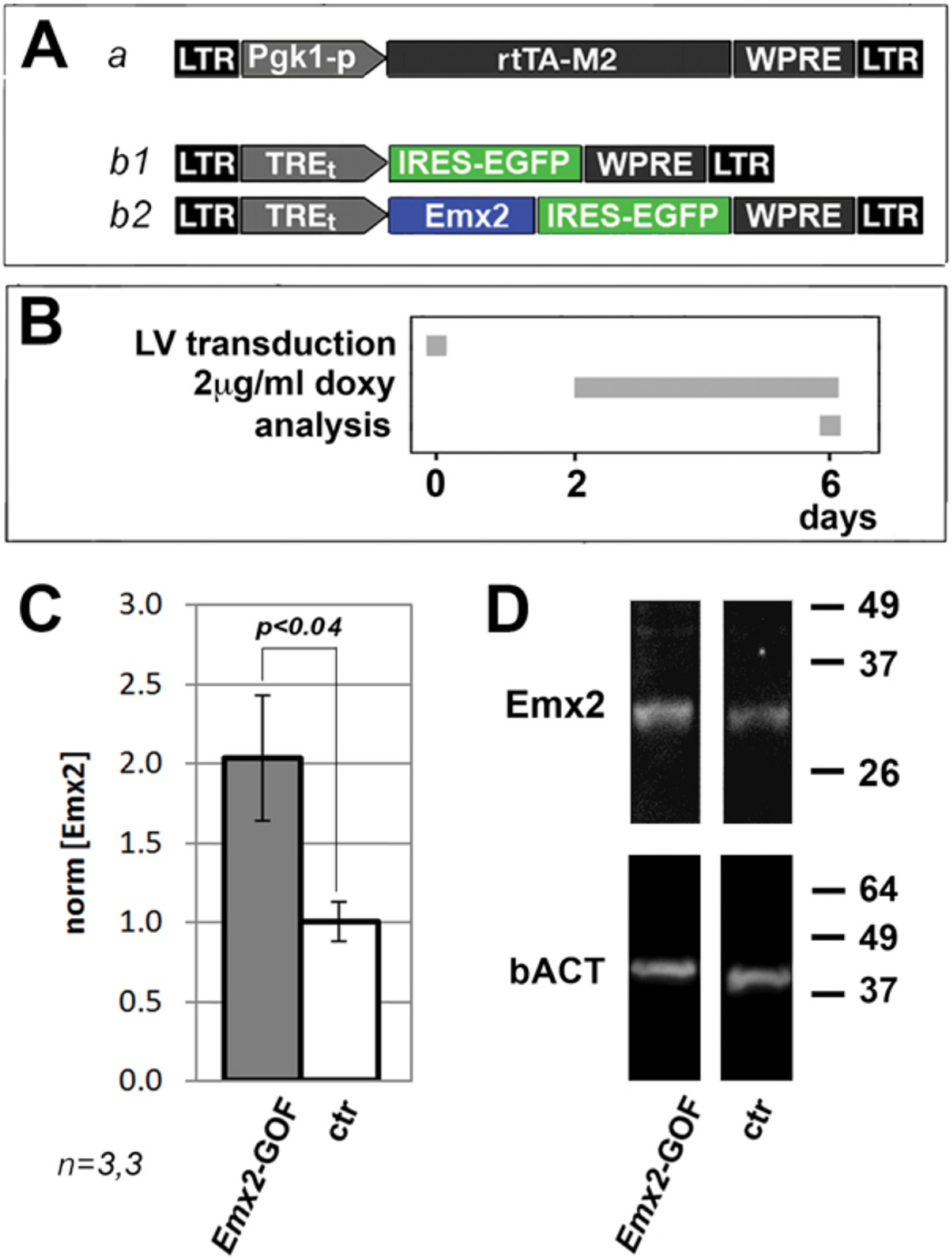

Supplementary Figure S1: Comparison of Emx2/EMX2 protein levels in U87MG cells in baseline conditions and upon *Emx2* overexpression. Here U87MG cell samples were engineered as in **A, B.** and profiled by western blot analysis as in **C, D** Values were normalized against controls. n is the number of biological replicates. p-value was calculated by t-test (one-tail, unpaired): $*p<0.05$.

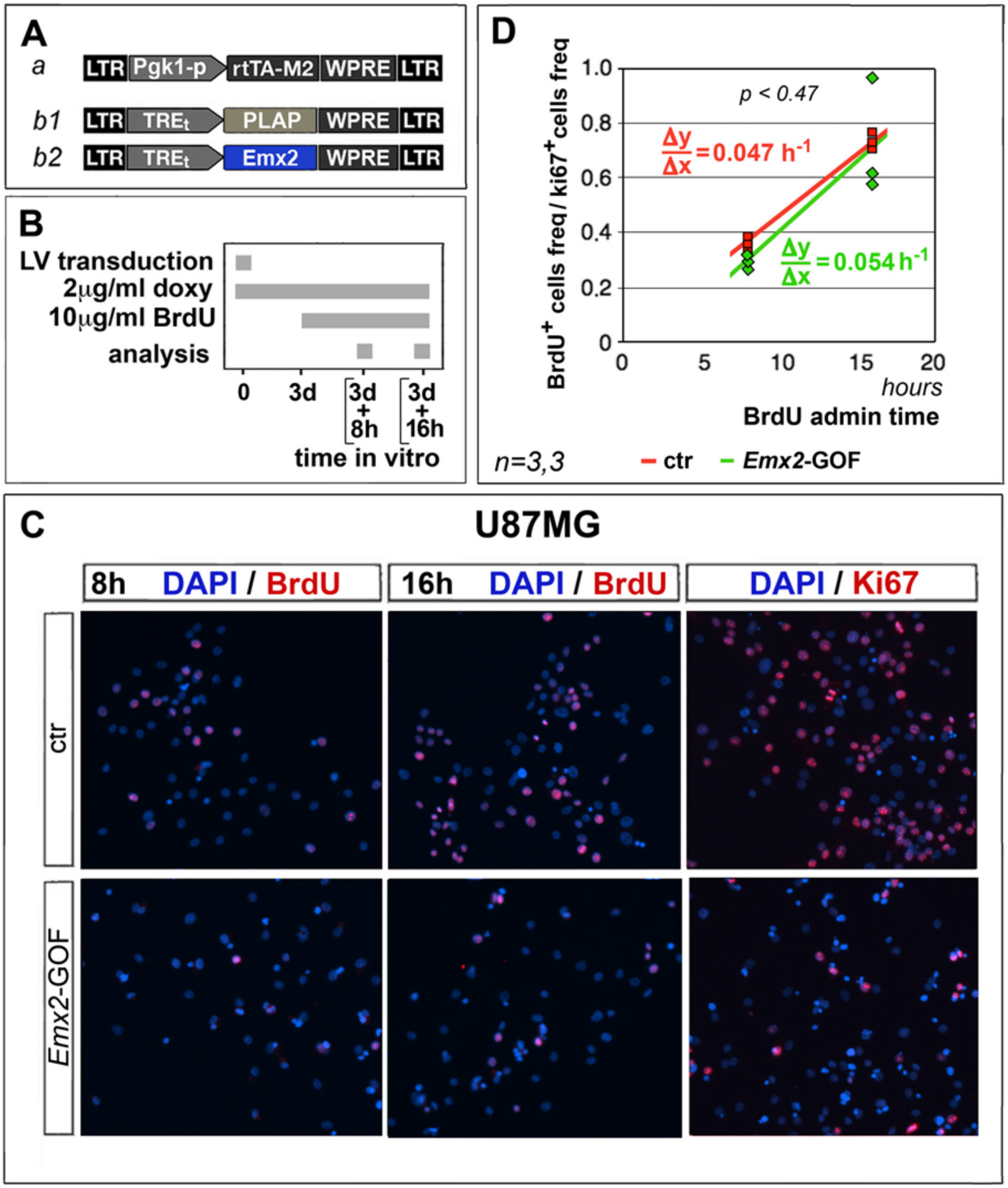

Supplementary Figure S2: Evaluation of G1-to-S phase progression of U87MG GBM cells upon *Emx2* overexpression. U87MG cells were engineered by lentiviral vectors and TetON technology as in **A.**, kept as adherent cultures and provided with doxycyclin and BrdU as in **B.** 8 or 16 hours after BrdU administration, cells were immunoprofiled for BrdU and further scored for Ki67 immunoreactivity **C.** Ki67$^+$ cell frequency-normalized BrdU$^+$ cell frequencies (y) were plotted against BrdU administration times (x). Slopes, Δy/Δx, representing the progression rate of G1 cells into S-phase, were calculated. Finally, statistical significance of their difference was evaluated by ANCOVA (one-way, unpaired) **D.** *n* is the number of biological BrdU replicates. Absolute frequencies of Ki67$^+$ cells were 0.538±0.026 and 0.197±0.003, in control and *Emx2*-GOF cultures, respectively (n=3,3; p<0.0001, by ANOVA, one-way, unpaired).

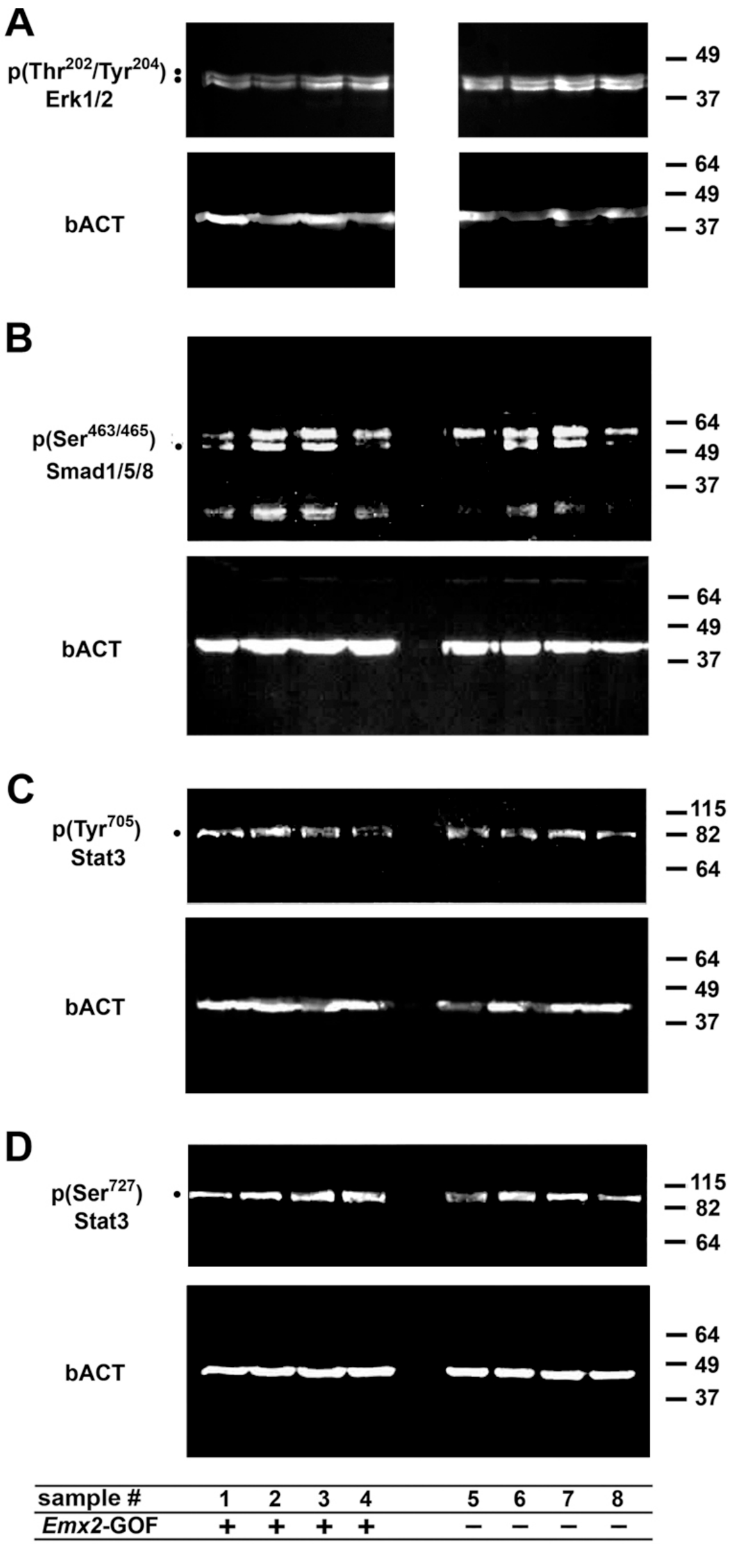

Supplementary Figure S3: Wider representation of western blots shown in Figure 2. This representation includes at least 6 bandwidths above and below the diagnostic band. In **C** and **D.** the top edge of the upper photograph corresponds to the slot line.

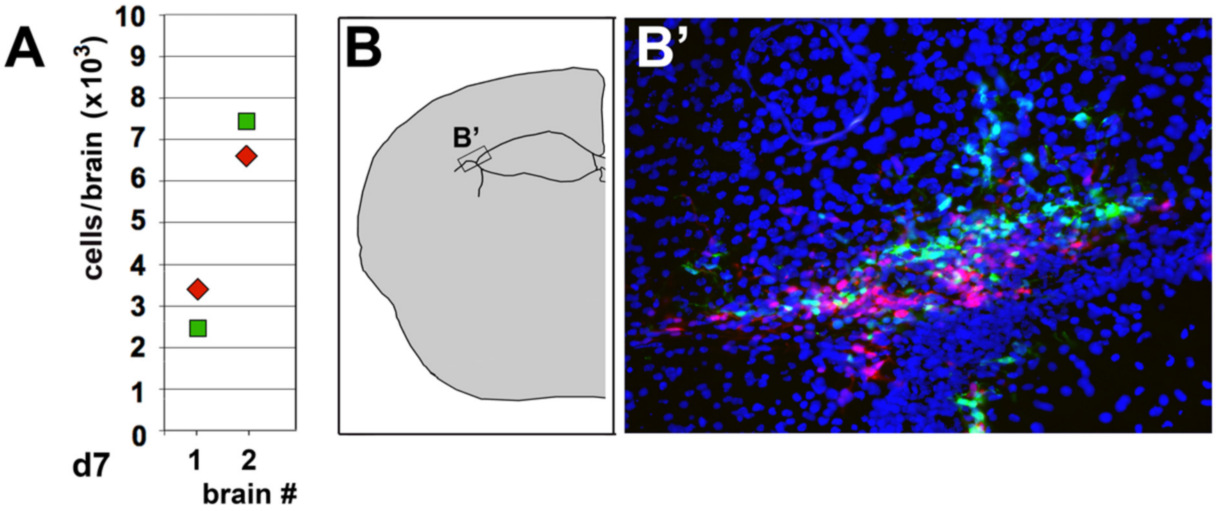

Supplementary Figure S4: Balanced survival of Egfp- and mCherry-labelled GBM cells upon co-transplantation into the neonatal cortical parenchyma. A-B'. The assay was run similar to Figure 4. Here cells, originating from GbmA line, were only transduced with constitutevely expressed fluoroprotein genes, mixed 1:1 and co-transplanted into 2 brains. *p*-value was calculated by t-test (one-tail, paired).

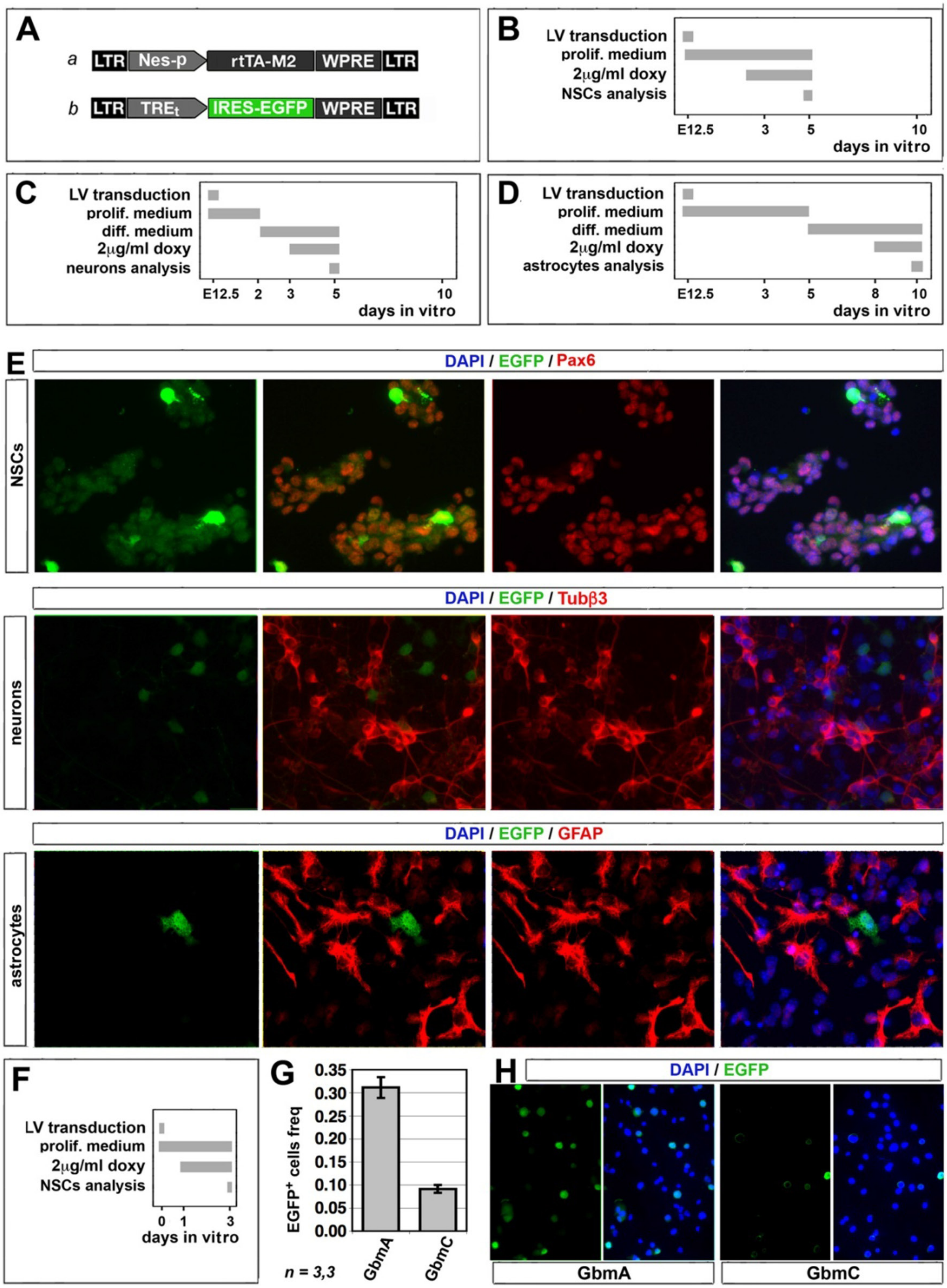

Supplementary Figure S5: Restriction of Nes-p promoter activity to embryonic neural stem cells and a subset of glioblastoma cells. E12.5 murine neural stem cells (NSCs) were engineered and cultured as shown in **A-D.** Cells were immunoprofiled at different days *in vitro* as shown in **B-D.**, for EGFP, driven by Nes-p promoter, and, alternatively, Pax6, Tubβ3 and GFAP **E.**. GbmA and GbmC glioblastoma cells were engineered and cultured as shown in **A, F.** and eventually immunoprofiled for EGFP **G, H.** *n* = number of biological replicates. bars = s.e.m.'s.

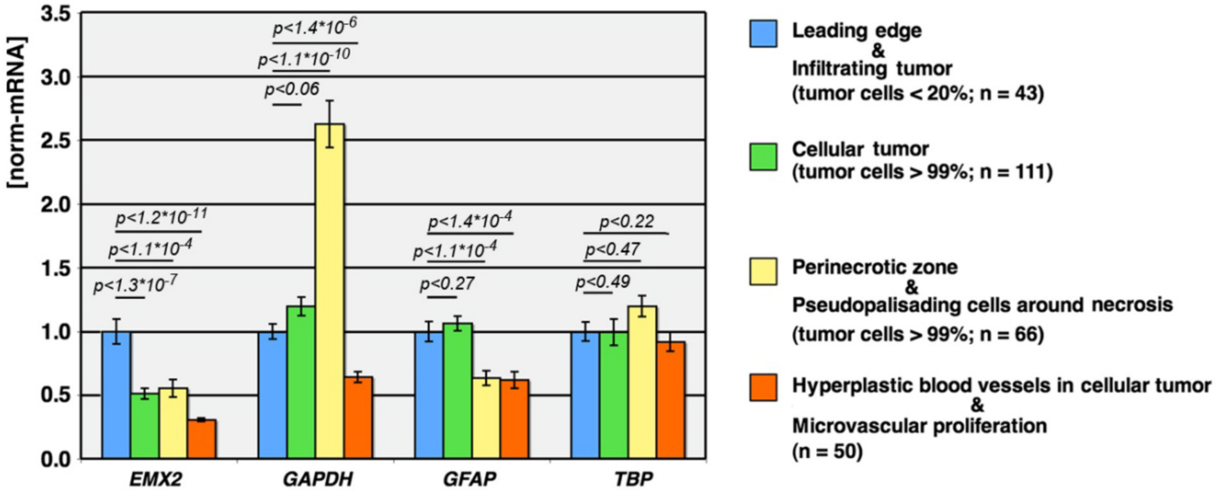

Supplementary Figure S6: Expression levels of *EMX2* and *GAPDH*, *GFAP* and *TBP* control genes in a set of human glioblastoma lesions. z-score-normalized RNASeq data, referring to (a) leading edge and infiltrating tumor, (b) cellular tumor, (c) perinecrotic zone and pseudopalisading cells around necrosis, and (d) hyperplastic blood vessels in cellular tumor and microvascular proliferation, were downloaded from the “Allen Brain Atlas - Ivy Glioblastoma Atlas Project” on 04.14.2016. They were averaged and statistically evaluated by t-test (one-way, unpaired). n = number of biological replicates. bars = s.e.m.'s.

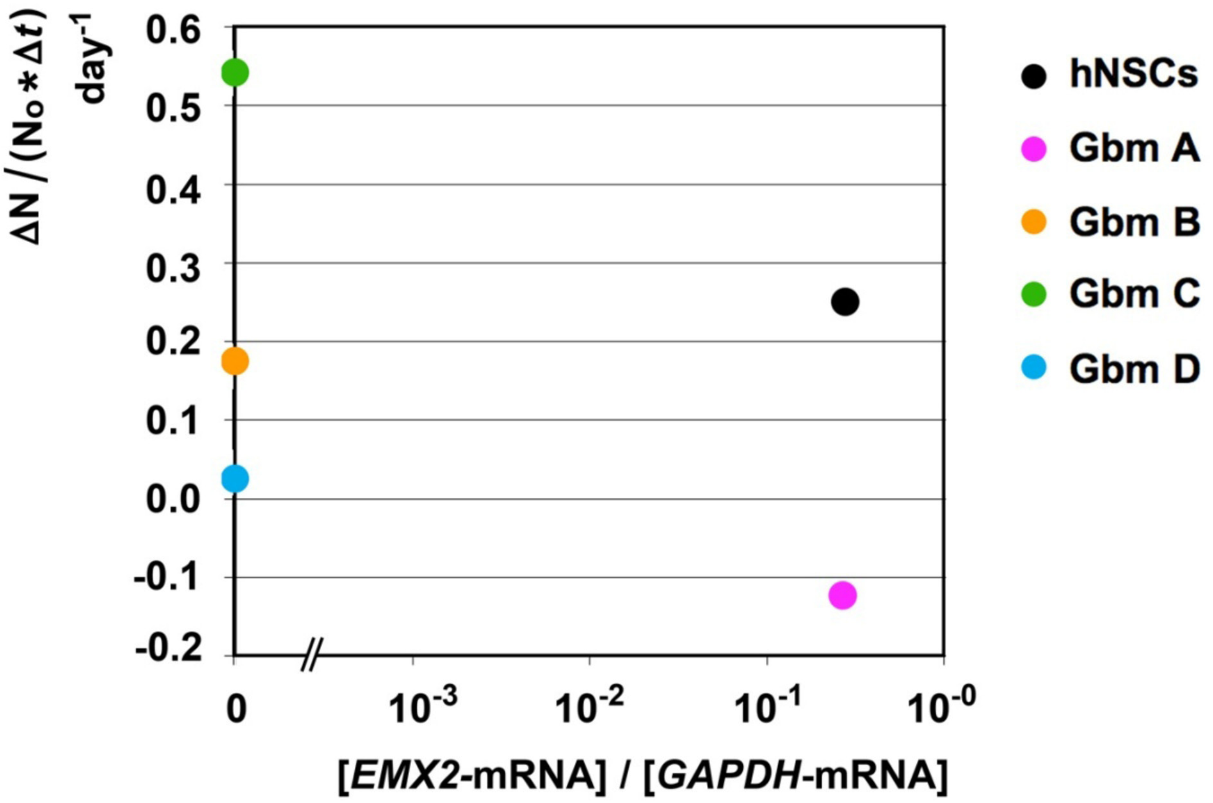

Supplementary Figure S7: Relationship between endogenous *EMX2*-mRNA levels and normalized expansion rate of distinct GBM cells. Here GBM culture growth was evaluated over the full times shown in Figure 1 and the $\Delta N/\Delta t$ expansion rates were normalized against the N_O initial number of cultured cells.

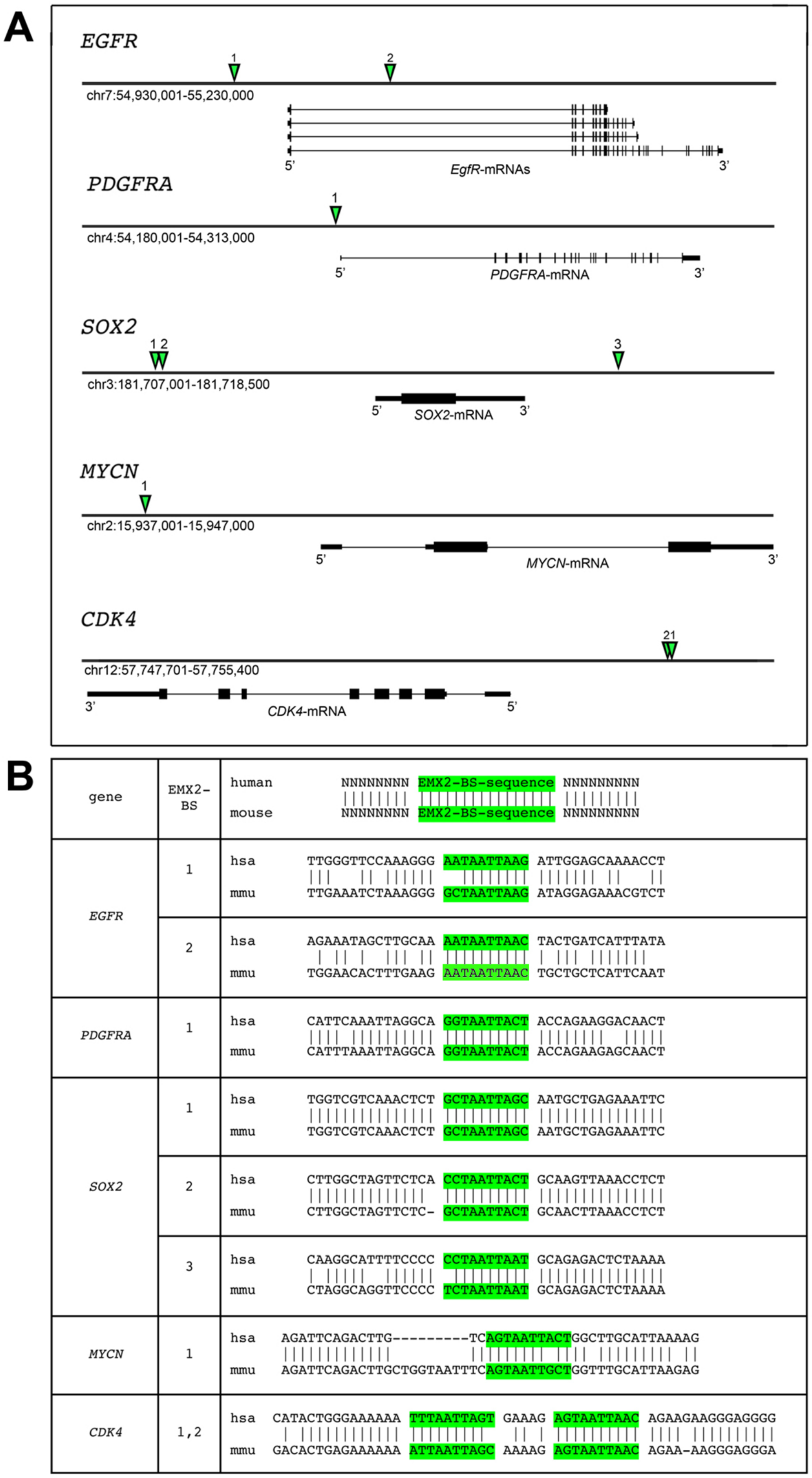

Supplementary Figure S8: In silico prediction of EMX2-binding sites in genes down-regulated by EMX2. A. Target genes and their surroundings were scanned by Jaspar software (http://jaspar.genereg.net/cgi-bin/jaspar_db.pl) for putative EMX2-binding sites, with relative profile score >0.95. Primary hits were further filtered for human/mouse conservation (by Blastn, according to the "Somewhat similar sequences" protocol) and mapped, as green arrowheads, to the corresponding human loci (as in the UCSC draft hg38). **B.** Sequences of putative human EMX2-binding sites and their murine counterparts referred to in (A).
